# Therapeutic potential of FAPI RLT in oncology: A systematic review

**DOI:** 10.7150/thno.106108

**Published:** 2025-03-10

**Authors:** Saad Ruzzeh, Ahmed Saad Abdlkadir, Diana Paez, Keon W. Kang, Tadashi Watabe, Serin Moghrabi, Andrew M. Scott, Akram Al-Ibraheem

**Affiliations:** 1 Department of Nuclear Medicine, King Hussein Cancer Center (KHCC), Amman, 11941, Jordan.; 2 Nuclear Medicine and Diagnostic Imaging Section, Division of Human Health, Department of Nuclear Sciences and Applications, International Atomic Energy Agency, Vienna, Austria.; 3 Department of Nuclear Medicine, Seoul National University College of Medicine, Seoul, Republic of Korea.; 4 Department of Biomedical Sciences, Seoul National University Graduate School, Seoul, Republic of Korea.; 5 Cancer Research Institute, Seoul National University, Seoul, Republic of Korea.; 6 Department of Nuclear Medicine, Seoul National University Hospital, Seoul, Republic of Korea.; 7 Department of Radiology, Graduate School of Medicine, Osaka University, Osaka 565-0871, Japan.; 8 Institute for Radiation Sciences, Osaka University, Osaka 565-0871, Japan.; 9 Department of Molecular Imaging and Therapy, Austin Health, 3084 Heidelberg, Australia.; 10 Department of Medicine, University of Melbourne, 1853 Melbourne, Australia.; 11 Olivia Newton-John Cancer Research Institute, and La Trobe University, 3084 Heidelberg, Australia.; 12 School of Medicine, The University of Jordan, Amman 11942, Jordan.

**Keywords:** [^177^Lu]Lu-FAPI, [^90^Y]Y-FAPI, Fibroblast activation protein inhibitor, FAPI RLT, Radionuclide therapy, Systematic review.

## Abstract

**Rationale:** This systematic review aims to examine the safety and efficacy of fibroblast activation protein inhibitor (FAPI) radioligand therapy (RLT) for various epithelial neoplasms.

**Methods:** PubMed, Web of Science, and Scopus databases were searched up to Jan 4, 2025, for studies involving FAPI RLT in various cancers. Data extraction focused on exploring safety and efficacy of FAPI RLT.

**Results:** Overall, 27 studies involving a total of 144 patients who received FAPI RLT were included in this systematic review. [^177^Lu]Lu-FAPI was employed in 21 studies, with 225 cycles administered to 95 patients at a median dose of 6.8 GBq/cycle. Six non-randomized clinical investigations using [^177^Lu]Lu-FAPI reported disease control rates ranging from 18.2% to 83.3%. Only three studies documented a cumulative total of six patients who experienced grade 3 or 4 toxicity post [^177^Lu]Lu-FAPI RLT. Of 16 case reports utilizing [^177^Lu]Lu-FAPI, nine achieved disease control across various cancer types, with no reported adverse events. Four studies employed [^90^Y]Y-FAPI, totaling 103 cycles in 42 patients at a median dose of 6.7 GBq/cycle. Three non-randomized clinical investigations reported disease control rates of 50% to 82%, with two studies documenting eight high-grade toxicity events. Furthermore, a successful administration of [^90^Y]Y-FAPI was employed in a single reported case involving multiple primary neoplasms with no reported adverse events. However, the patient did not achieve disease control post [^90^Y]Y-FAPI. A cohort study utilized 53 [^213^Bi]Bi-FAPI-46 injections following a fractionated dose regimen in six cancer patients, achieving a 33.3% disease control rate without reported adverse events. One case report described dual radionuclide therapy using two cycles with a cumulative 20 GBq [^153^Sm]Sm-FAPI and a third 8 GBq [^90^Y]Y-FAPI cycle in a lung cancer patient, resulting in stable disease for eight months.

**Conclusion:** FAPI RLT is a promising and safe therapeutic agent in oncology, with potential benefits achieved on short-term basis. However, its long-term efficacy and safety require further research with larger, controlled studies, considering the currently observed variations in patient populations, cancer types, and methodologies within reviewed studies.

## Introduction

Fibroblast activation protein (FAP), a member of the serine protease family, has emerged as a significant target in oncology because of its high expression in the stroma of more than 90% of epithelial malignancies, including breast, colorectal, lung, ovarian and pancreatic cancers 1-5. FAPs are predominantly found in cancer-associated fibroblasts (CAFs), which are integral to the tumor microenvironment (TME) and play crucial roles in tumor progression, metastasis and immune regulation 6. Given its limited expression in normal tissues, FAP is an attractive target for both imaging and therapeutic purposes, particularly in the context of theranostics, where diagnostic and therapeutic capabilities are combined 7.

Recent advancements have led to the development of various FAP inhibitors (FAPIs) that have been labeled with radionuclides for positron emission tomography/computed tomography (PET/CT) imaging. FAPI-labeled radiopharmaceuticals, such as [^68^Ga]Ga-FAPI, have shown significant uptake in FAP-positive tumors, providing a powerful tool for cancer diagnosis 1. Numerous systematic reviews and meta-analyses have revealed that FAPI PET imaging often outperforms traditional [^18^F]FDG and other non-FAPI PET tracers in certain cancers, including gastric, pancreatic, breast, and lung cancers, and rare neoplasms 8-13, owing to its higher specificity and lower background uptake. This superior imaging performance makes FAPI PET a promising modality for more accurate tumor detection and staging in oncology 14.

In addition to its diagnostic capabilities, FAPI has also been explored as a therapeutic agent in the form of radioligand therapy (RLT). Radiolabeled FAPIs, such as [^177^Lu]Lu-FAPI, [^90^Y]Y-FAPI, and [^225^Ac]Ac-FAPI, have demonstrated the ability to deliver targeted radiotherapy to FAP-expressing tumors 15, 16. These therapies are particularly promising in cancers such as breast, thyroid and pancreatic cancer, where FAP expression is prevalent in the TME 17. By selectively targeting FAP, these treatments have the potential to minimize damage to surrounding healthy tissues and improve patient outcomes.

This systematic review aims to provide a comprehensive analysis of the theranostic potential of FAPI RLT across various cancer types, highlighting the current state of research, the efficacy of FAP-targeted therapies and the challenges that need to be addressed to fully realize the potential of FAPI-based theranostics in oncology.

## Materials and Methods

This systematic review adhered to the Preferred Reporting Items for Systematic Reviews and Meta-Analyses (PRISMA) guidelines (Supplementary Table 1) 18, and has been registered with PROSPERO (Registration ID: CRD42024614776).

### Search strategy

PubMed, Web of Science and Scopus were systematically searched from inception till 4^th^ Jan 2025 utilizing focused key terms relevant to the topic of interest (Supplementary Table 2).

### Eligibility criteria

Original research articles, clinical trials, case series and case reports focusing on clinical in-human FAPI RLT applications were included. Studies involving patients diagnosed with any type of cancer treated with any FAPI RLT agent were considered. Review articles, editorials, conference abstracts, commentaries, animal studies and incomplete studies, were excluded. Only studies published in English language were retrieved and examined to ensure the accuracy and consistency of the data extracted.

### Screening and data extraction

Two authors conducted the initial screening by evaluating the titles and abstracts of the records in Rayyan AI 19. Next, they independently performed a secondary screening by thoroughly reviewing the full texts of the identified studies on the basis of predetermined inclusion criteria. The authors subsequently independently extracted data from the included studies via a predesigned Microsoft Office Excel Professional Plus 2016. The extracted data encompassed various aspects, such as study design, patient demographics, type of cancer, specific molecular imaging modalities used, previous treatment, dosage and type of FAPI RLT administered, number of administered cycles, clinical outcomes and imaging response according to the Response Evaluation Criteria in Solid Tumors Version 1.1 (RECIST 1.1) and the PET Response Criteria in Solid Tumors (PERCIST) criteria.

The reported adverse effects or toxicities according to the Common Terminology Criteria for Adverse Events (CTCAE) were also extracted. This criterion is a standardized system developed by the National Cancer Institute (NCI) to classify and grade the severity of adverse events (AEs) in patients undergoing cancer therapy. This system provides consistent terminology and a grading scale from 1 to 5, where Grade 1 indicates mild events, and Grade 5 represents fatal outcomes 20.

The extracted data were reviewed and validated by the reviewers to ensure accuracy and completeness. Disagreements during the data extraction process were resolved through discussion, and a third reviewer was consulted if necessary.

### Risk of bias and quality assessment

Two researchers assessed the risk of bias in each clinical study included in the systematic review via the Risk of Bias in Nonrandomized Studies - of Interventions (ROBINS-I) tool 21. Each domain was assessed individually with judgments categorized as low, moderate, serious, critical risk of bias, or no information, depending on the study design.

For each domain, signaling questions were used to guide the judgment, and the overall risk of bias for each study was determined on the basis of the answers provided. The assessment was conducted via Excel, and its graphical representation was created via robvis 22 with an additional reviewer brought in to resolve any potential disagreements between the primary reviewers.

## Results

### Study selection

**Figure 1:**
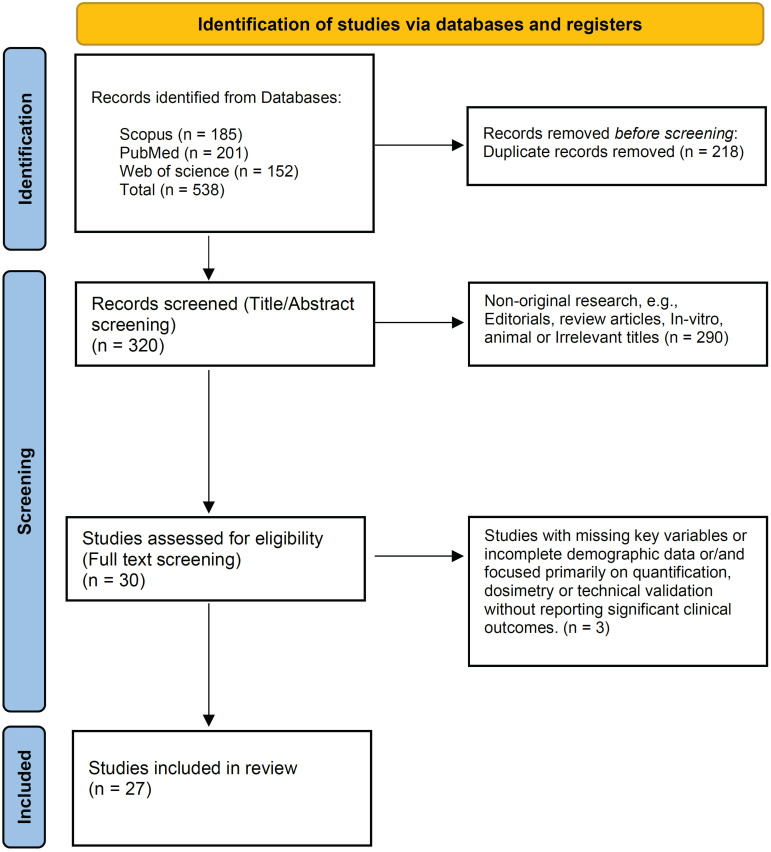
The selection process using the Preferred Reporting Items for Systematic Reviews and Meta-Analyses (PRISMA) flow diagram.

The initial search identified 538 published articles, from which 218 duplicates were eliminated, leaving 320 records. Upon screening titles and abstracts, 290 studies were excluded. With full-text screening, 3 studies were excluded. Finally, 27 studies (10 original studies and 17 case reports) were included in the review (Figure 1) 23-49.

### Characteristics of the included studies

Table 1 and supplementary Table 3 summarize the study-based analysis of the included studies exploring the use of FAPI RLT in various cancers.

**Figure 2:**
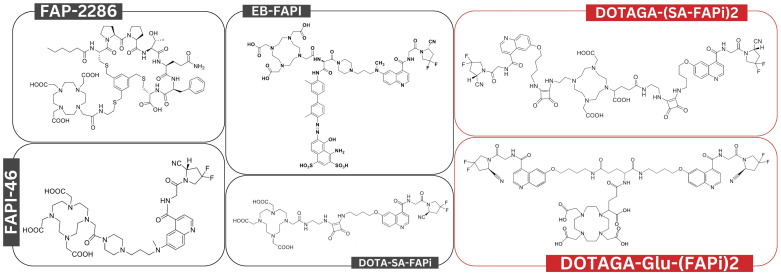
Overview of chemical structures for various FAPI RLT agents examined in this systematic review. Chemical structures labeled in red represent bivalent FAPI RLT, while those labeled in black are monovalent.

The studies included a total of 144 patients who received FAPI RLT. These were conducted in Iran, Germany, China, India, and United States and investigated various cancer types, including breast, thyroid, pancreatic, and lung cancer. The sample sizes ranged from 1 to 21 patients, with a median age of 52 years. [^90^Y]Y-FAPI-46, [^153^Sm]Sm-FAPI-46, and [^213^Bi]Bi-FAPI-46 alongside various [^177^Lu]Lu-FAPI RLT agents were used (Figure 2 and Supplementary Table 4).

### Risk of bias and quality assessment

**Figure 3:**
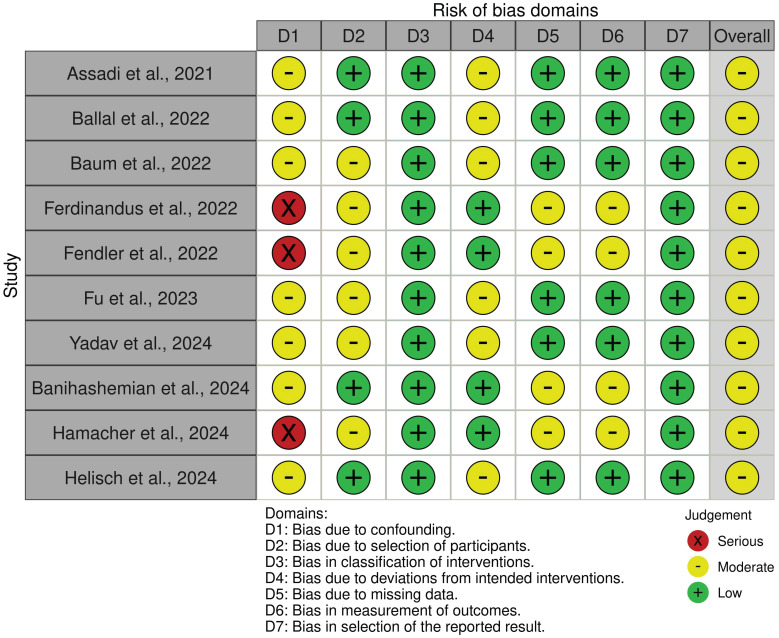
Risk of Bias in Nonrandomized Studies - of Interventions (ROBINS-I) Assessment for Included Studies.

In this review, the risk of bias and quality assessment of the 10 included clinical studies via the ROBINS-I tool predominantly demonstrated moderate to low risk across most domains. Only three studies displayed serious bias risk due to confounding 46-48. Moderate bias risk was observed in all remaining studies primary stemming from deviations from intended interventions, missing data, measurement of outcomes and selection of the reported results 23, 25, 29, 32, 34, 41, 44, 46-48. Figure 3 and Supplementary Table 5 outline the domains affected by bias and provide a rationale for risk of bias identified in certain domains of the ROBINS-I tool.

## [^177^Lu]Lu-FAPI RLT: Systematic Review

This systematic review identified a total of 21 studies utilizing a total of 225 [^177^Lu]Lu-FAPI RLT cycles offered at a median active dose of 6.8 GBq/cycle 23-43. These studies explored [^177^Lu]Lu-FAPI RLT utility in various cancer types encompassing a total of 95 patients (38 males and 57 females). Table 2 outlines etiology-based analysis of studies utilizing [^177^Lu]Lu-FAPI RLT.

Among the patients with head and neck malignancies, 2 patients had CNS tumors, 1 patient had nasopharyngeal carcinoma, and 30 patients had thyroid cancer. Thoracic malignancies were explored in 30 patients having breast cancer and 3 patients having lung cancer. For gastrointestinal malignancies, 8 patients had pancreatic cancer, and 4 had colorectal cancer. The patients with genitourinary malignancies included 1 patient each with urinary bladder cancer and prostate cancer. The gynecological malignancies were detected in 3 patients with ovarian cancer and 1 patient with cervical cancer. Among the patients with mesenchymal malignancies, 7 patients had sarcoma, and 2 had solitary fibrous tumors. Additionally, rare tumors were represented by 1 patient with a round cell tumor and 1 patient with MEN2A (paraganglioma/pheochromocytoma). A qualitative analysis based on these cancer types is presented in the following sections.

Importantly, before proceeding further in this review, most of the included studies reported imaging responses according to the RECIST 1.1 and PERCIST criteria. However, a few studies did not report imaging outcomes using these standardized criteria. In such cases, we derived conclusions on the basis of the provided images and the authors' reported findings.

### Head and neck malignancies: thyroid cancer

Assadi *et al.* conducted a preliminary study including 18 patients with different types of cancer 23. Among these patients, a 70-year-old male patient with anaplastic thyroid cancer had metastasized to regional lymph nodes and bones. After surgery, radiotherapy, and chemotherapy, the patient received one cycle of [^177^Lu]Lu-FAPI-46 therapy at a dose of 3.7 GBq. Follow-up imaging was performed 6-8 weeks after therapy, and the response was evaluated according to RECIST 1.1, which indicated SD. No significant adverse effects or toxicities were reported 23.

One year later, Ballal *et al.* conducted an exploratory study in India to assess the clinical safety and efficacy of [^177^Lu]Lu-DOTAGA-(SA-FAPi)_2_ in patients with relapsed or refractory differentiated thyroid cancer who had progressed on tyrosine kinase inhibitors 25. The study enrolled 15 patients (11 females, 4 males) who received intravenous [^177^Lu]Lu-DOTAGA-(SA-FAPi)_2_ at eight-week intervals. The primary endpoints were thyroglobulin response and functional imaging response. Toxicities were graded using CTCAE version 5.0. A total of 45 cycles were administered, with a median cumulative administered activity of 8.2 GBq (interquartile range: 5.5-14 GBq). Serum thyroglobulin levels decreased significantly post-treatment [(median Tg: baseline 10,549 ng/mL (interquartile range: 3066.5-39,450) vs. assessment time 5649 ng/mL (interquartile range: 939.5-17,099), *p* = 0.0005)]. Molecular response assessment revealed no complete responses; however, partial responses (PR) were documented in four patients, and SD was evident in three. All remaining patients were not assessed with posttreatment molecular imaging. No patients experienced high-grade toxicity. One patient experienced grade I diarrhea after each treatment cycle, lasting up to 24 hours and managed with oral hydration. Three patients with baseline grade I fatigue reported increased fatigue lasting up to three days post-treatment 25.

Ballal *et al.* reported a 56-year-old male with metastatic medullary thyroid carcinoma 26. The patient was treated with [^177^Lu]Lu-DOTAGA-(SA-FAPi)_2_ after initial surgery, chemotherapy and radiotherapy. [^68^Ga]Ga-DOTA.SA.FAPI PET/CT was used for imaging. The patient received a single dose of 1.65 GBq. At the six-week follow-up, there was a notable improvement in quality of life and a downward trend in chromogranin A (CgA) levels, with a PR observed. Blood parameters monitored every 2 weeks did not indicate any Grade III/IV toxicity. Tumor retention was sufficiently prolonged, even up to 168 hours posttreatment 26.

Fu *et al.* explored the safety and efficacy of [^177^Lu]Lu-FAPI-46 in a 34-year-old male with radioiodine-refractory differentiated thyroid cancer 33. Prior to treatment, the patient had undergone surgery and radioiodine therapy. [^68^Ga]Ga-FAPI-46 PET imaging was used for assessment, and the patient then received 4 cycles of [^177^Lu]Lu-FAPI-46, with a cumulative dose of 16.7 GBq. During treatment, analgesic scores decreased from 3 to 1, and the Eastern Cooperative Oncology Group (ECOG) performance status improved from 1 to 0. Posttreatment laboratory parameters, including complete blood cell count, liver, and renal function, remained within normal ranges. No therapy-related adverse symptoms were reported. Imaging at 6-8 weeks after therapy revealed SD 33.

Fu *et al.* also conducted an open-label, nonrandomized, first-in-human, dose-escalation trial to evaluate the safety and efficacy of [^177^Lu]Lu-EB-FAPI ([^177^Lu]Lu-LNC1004) in patients with metastatic radioiodine-refractory thyroid cancer 34. The study involved 12 patients (8 males and 4 females), one with medullary thyroid cancer, one with follicular thyroid cancer and the remainder with papillary thyroid cancer. The 6-week treatment cycle initially used a dose of 2.22 GBq, and subsequent cohorts received incremental 50% increases until dose-limiting toxicity (DLT) was observed. The treatment was well tolerated, with no life-threatening adverse events and no DLTs in the initial cohort. One patient experienced Grade 4 thrombocytopenia, and two patients experienced Grades 3 and 4 hematotoxicity. Using the RECIST 1.1 criteria, the study revealed a 25% objective response rate and an 83% disease control rate, with 3 patients achieving a PR, 7 showing SD, and 2 having progressive disease (PD) 34.

### Other head and neck malignancies

Ballal *et al.* explored the safety and efficacy of [^177^Lu]Lu-DOTAGA-Glu-(FAPI)_2_ in a 52-year-old female with glioblastoma multiforme 27. Baseline [^68^Ga]Ga-FAPI PET/CT imaging was offered and revealed intense [^68^Ga]Ga-FAPI expression at regions of interest. The patient received two cycles of [^177^Lu]Lu-DOTAGA-Glu-(FAPI)_2_ totaling 14.8 GBq. The [^68^Ga]Ga-FAPI PET/CT imaging response at 6-9 weeks posttherapy indicated a PR with an improvement in symptoms and performance status, with no adverse events observed during the treatment or follow-up 27.

Wan *et al.* reported a 43-year-old male with rhabdoid meningioma that metastasized to the liver, pancreas, and bones 40. The patient underwent [^68^Ga]Ga-FAPI-2286 PET/CT imaging before receiving one cycle of [^177^Lu]Lu-FAPI-2286 at a dose of 7.4 GBq. The imaging response at 6-9 weeks posttherapy was a PR, with no adverse symptoms reported 40.

Fu *et al.* treated a 25-year-old male with nasopharyngeal cancer and metastases to the lymph nodes, liver, and bones 35. The patient had undergone surgery and received chemotherapy before receiving one cycle of [^177^Lu]Lu-FAPI-46 therapy at a dose of 3.7 GBq. Three days after treatment, the patient reported reduced ostealgia. However, the imaging response at 6-9 weeks posttreatment revealed PD, with no therapy-related adverse effects noted 35.

### Thoracic malignancies: breast cancer

A total of five studies included in this systematic review discussed the use of [^177^Lu]Lu-FAPI in patients with breast cancer. These studies involved 30 patients, 29 females and 1 male.

Ballal *et al.* presented a 31-year-old female patient with metastatic breast cancer of invasive ductal carcinoma histopathology 24. The cancer had spread to regions such as the lungs, liver, bones, and brain. The patient then received a therapeutic dose of 3.2 GBq of [^177^Lu]Lu-DOTA-SA-FAPI for compassionate care. Post-lutetium scintigraphy was performed 24 hours after the administration of the radiopharmaceutical. Posttreatment, the patient reported a decrease in the intensity of headaches, and her laboratory parameters remained within normal limits at the 4-week follow-up. Notably, no treatment-related adverse events were observed. However, follow-up PET or conventional imaging was not conducted or reported in this study 24.

The preliminary study conducted in by Assadi *et al.* included five female patients with metastatic breast cancer 23. All patients had a history of surgery, radiotherapy, and chemotherapy, with metastases in the bones, liver, lymph nodes, peritoneum, lungs, and pleura. The mean age was 45 ± 7.2 years. Each patient underwent [^68^Ga]Ga-FAPI-46 PET/CT imaging and received a median of 2 cycles (range: 1-3) of [^177^Lu]Lu-FAPI-46, with a median cumulative dose of 7.4 GBq (range: 1.85-12.95 GBq). Subjective scale responses indicated that 33.33% of patients had moderate responses and that 66.66% had poor responses. Follow-up assessments revealed that 66.66% of patients had stable disease, and 33.33% had progressive disease. Conventional imaging via RECIST 1.1 criteria at 6-8 weeks posttreatment confirmed SD in 3 patients and 2 PD patients. No adverse effects or toxicities were reported 23.

Baum *et al.* evaluated four female patients with breast cancer (out of 11 patients in the study with various cancers) with a median age of 58 years following [^177^Lu]Lu-FAP-2286 RLT 32. All had previously undergone surgery, radiotherapy, hormonal therapy, and, in some cases, additional treatments such as human epidermal growth factor 2 (HER2) and bisphosphonate. They received 2 cycles of [^177^Lu]Lu-FAP-2286, with a mean cumulative dose of 5.8 GBq (range: range, 2.4-9.9 GBq). Posttherapy evaluation according to RECIST 1.1 in all patients at 6-8 weeks revealed that two patients had PD, whereas two patients had SD. The adverse effects reported included grade 3 (G3) leukopenia (preexisting G2), G2 anemia (from preexisting G1), G3 pancytopenia (from preexisting 1-2) and G2 abdominal pain 32.

Yadav *et al.* evaluated 19 breast cancer patients (18 females and 1 male) with a median age of 50 years 41. All patients had previously undergone chemotherapy, radiotherapy, and hormonal therapy. Baseline imaging was performed utilizing [^68^Ga]Ga-DOTA-SA-FAPI PET/CT. They received a median of 3 cycles (range: 2-6) of [^177^Lu]Lu-DOTA-SA-FAPI dimers, with a median injected activity of 5.5 GBq per cycle (range: 5.1-5.7 GBq) and a mean cumulative activity of 19 GBq (range: 11-33.3 GBq). Treatment outcomes based on visual analog scale criteria revealed that 26.3% of patients achieved a complete response, 15.7% had a partial response, 42% had a minimal response, 11% had stable disease, and 5% had no response. The clinical overall response rate was 84%, and the clinical disease control rate was 95%. Follow-up PET/CT scans revealed that 25% of patients achieved PR and 37.5% had PD compared with baseline. Adverse effects included new-onset anemia in 3 patients (grade 1 in 2 patients and grade 2 in 1 patient), whereas preexisting anemia and thrombocytopenia did not worsen. There were no significant changes in renal parameters or severe toxicities. The median follow-up period was 14 months, with a median overall survival of 12 months and a median progression-free survival of 8.5 months 41.

Banihashemian *et al.* explored combined therapeutic approach involving both chemotherapy and [^177^Lu]Lu-FAP-2286 in a 42-year-old female breast cancer patient with lymph node, bone and skin metastases 28. Owing to the missed opportunity for surgical intervention, the patient received six cycles of Docetaxel, Carboplatin, Trastuzumab, and Pertuzumab injections at three-week intervals. Additionally, after 13 days, four cycles of 6.6 GBq [^177^Lu]Lu-FAP-2286 were concurrently administered at six-week intervals, resulting in a total cumulative dose of 26.6 GBq. Imaging with [^68^Ga]Ga-FAP-2286 PET/CT was used to evaluate treatment response, which was assessed according to the PERCIST criteria. The patient achieved a complete molecular response (CR) without experiencing any Grade 3 or 4 adverse events during the follow-up period 28.

### Thoracic malignancies: lung cancer

Three included studies evaluated the use of [^177^Lu]Lu-FAPI in three male patients with lung cancer. Assadi *et al.* explored the use of [^177^Lu]Lu-FAPI-46 in a 55-year-old male patient with lung adenocarcinoma 23. The patient had regional, lymph node, bone and pleural metastases and received chemotherapy. The patient received four cycles of [^177^Lu]Lu-FAPI-46 therapy with a cumulative dose of 13.7 GBq. The clinical response was SD. Notably, no adverse events or toxicities were reported 23.

Rao *et al.* investigated the use of [^177^Lu]Lu-FAP-2286 in a 49-year-old male patient with squamous lung cancer and pleural, lymph node, liver, and bone metastases who had previously undergone surgery 39. Baseline imaging via [^68^Ga]Ga-FAP-2287 PET/CT was offered prior to [^177^Lu]Lu-FAP-2286 RLT to determine eligibility. The patient received one cycle of [^177^Lu]Lu-FAP-2286 therapy at a dose of 7 GBq. Posttherapy imaging revealed a significant decrease in the number and degree of tracer uptake in the lesions, indicating PR. No adverse reactions or toxicities were observed 39.

Yang *et al.* assessed the use of [^177^Lu]Lu-FAPI-2286 in a 56-year-old male patient with lung adenocarcinoma 42. The patient had metastases in the lymph nodes and brain and was scheduled to receive [^177^Lu]Lu-FAPI-2286 therapy combined with immunotherapy. Pretreatment imaging was performed via [^68^Ga]Ga-FAP-2286 PET/CT. The patient received one cycle of [^177^Lu]Lu-FAPI-2286 therapy at a dose of 7.4 GBq. Half a month later, the patient experienced significant relief from dyspnea. Imaging results revealed a positive therapeutic response, with notable reductions in tracer uptake, lesion size, and number across multiple sites. Specifically, the right lung soft tissue mass, lymph node metastases, and bilateral lung metastases all presented decreased maximum standardized uptake value (SUVmax) values, and the brain lesion had completely disappeared. In other words, the imaging response of the PR was achieved. The patient did not report any adverse effects. The authors concluded that their limited experience suggested the potential for [^177^Lu]Lu-FAPI-2286 radiation therapy combined with targeted therapy, although further research is needed 42.

### Gastroenteropancreatic malignancies: pancreatic cancer

Three studies reported the use of [^177^Lu]Lu-FAPI in patients with pancreatic cancer, involving 8 patients (3 females and 5 males). Assadi *et al.* 2021 preliminarily [^177^Lu]Lu-FAPI-46 therapy study assessed two patients, a 79-year-old male and a 50-year-old female, with metastatic pancreatic cancer 23. Both patients, who had extensive metastatic sites and had previously undergone chemotherapy, underwent [^68^Ga]Ga-FAPI-46 PET/CT imaging followed by [^177^Lu]Lu-FAPI-46 therapy. The first patient received a cumulative dose of 5.55 GBq over two cycles, and the second patient received 3.7 GBq in one cycle. Posttherapy imaging, conducted 6-8 weeks after treatment and assessed according to the RECIST 1.1 criteria, indicated PD in both patients, with no reported adverse events 23.

Kaghazchi *et al.* presented an interesting case involving a 52-year-old female patient with end-stage pancreatic adenocarcinoma who had metastases to the liver, lymph nodes, bone, and lung 36. After previous treatments, including surgery and chemotherapy, she underwent [^68^Ga]Ga-FAPI-46 PET/CT imaging followed by a single cycle of 1.85 GBq [^177^Lu]Lu-FAPI-46 therapy. No adverse events were reported, but posttherapy SPECT/CT conducted daily for six days showed limited therapeutic benefit due to rapid radiotracer washout and tumor progression. However, clinically, the patient experienced a transient reduction in right shoulder pain after one week, but the pain and tumor marker levels increased after two weeks. Unfortunately, the patient passed away 45 days posttreatment, before imaging response assessment by PET/CT or conventional imaging, and no adverse events were reported 36.

Baum *et al.* conducted a retrospective clinical study involving five patients with pancreatic cancer, three of whom presented with metastases to various sites, including the lymph nodes, liver, peritoneum, and bone; the remaining two patients were therapy naïve 32. The patients, aged between 55 and 87 years, underwent [^68^Ga]Ga-FAP-2286 or [^68^Ga]Ga-FAPI-04 PET/CT imaging, followed by two cycles of [^177^Lu]Lu-FAP-2286 therapy with a cumulative dose of approximately 5.8 GBq. Posttherapy scans were performed daily for 7-10 days. One of the patients reported significant pain relief and reduced morphine use, whereas the other experienced improvements in physical capacity, quality of life, and pain relief. Imaging response according to the RECIST 1.1 criteria revealed PD in all patients. No significant adverse events were reported 32.

### Gastroenteropancreatic malignancies: colorectal cancer

Two included studies evaluated the use of [^177^Lu]Lu-FAPI in patients with colorectal cancer. These studies included a total of 4 patients, 3 of whom were male. Assadi *et al.* evaluated the use of [^177^Lu]Lu-FAPI-46 in three patients (2 males and one female) with advanced colon cancer with a range of metastatic sites, including the liver, lymph nodes, bone and lung 23. The patients had previously undergone various treatments, including surgery, chemotherapy, and radiotherapy. Each patient was evaluated with [^68^Ga]Ga-FAPI-46 PET/CT before receiving [^177^Lu]Lu-FAPI-46 therapy. One patient received two cycles with a cumulative dose of 7.4 GBq, whereas the others received one cycle with a cumulative dose of 3.7 GBq. Posttherapy imaging was performed 24 hours after treatment. The imaging responses varied: SD was observed in two patients, whereas one patient experienced PD. No significant adverse events were reported 23.

Baum *et al.* explored the application of [^177^Lu]Lu-FAP-2286 in a 61-year-old male patient with metastatic rectal cancer who presented with metastases to the liver, lungs, and lymph nodes 32. The patient had previously undergone surgery and received both radiotherapy and chemotherapy. As part of the study cohort, pretreatment imaging was performed via either [^68^Ga]Ga-FAP-2286 or [^68^Ga]Ga-FAPI-04 PET/CT. The patient was administered a single cycle of [^177^Lu]Lu-FAP-2286 therapy. To evaluate treatment efficacy, follow-up imaging—including CT or magnetic resonance imaging (MRI) and [^68^Ga]Ga-FAP-2286 PET/CT—was conducted at 6-8 weeks posttherapy. The results indicated PD, and the patient experienced Grade 2 anemia as an adverse event 32.

### Genitourinary malignancies

Assadi *et al.* evaluated the use of [^177^Lu]Lu-FAPI-46 in a 65-year-old male patient with prostate cancer and bone metastases who was receiving androgen deprivation therapy received a single cycle of [^177^Lu]Lu-FAPI-46 at a dose of 1.85 GBq 23. Imaging conducted 6-8 weeks after treatment revealed SD. No [^177^Lu]Lu-FAPI-46-induced toxicity reported 23.

Li *et al.* presented a 73-year-old male with urinary bladder cancer that had metastasized to the liver, kidneys, and adrenal glands 37. The patient was imaged with [^68^Ga]Ga-FAP-2286 PET/CT and subsequently received one cycle of [^177^Lu]Lu-FAP-2286 at a dose of 7.4 GBq. During follow-up, the patient experienced significant symptom relief. Three months after treatment, a follow-up [^68^Ga]Ga-FAP-2286 PET/CT scan for response evaluation revealed a marked reduction in both the number and intensity of tracer uptake in the lesions indicating PR. No adverse reactions or toxicities were observed 37.

### Mesenchymal malignancies

Five studies were conducted on the use of [^177^Lu]Lu-FAPI in patients with mesenchymal malignancies. These studies included a total of 9 patients, of which 4 were female and 5 were male. In his preliminary study, Assadi presented a 6-year-old male patient with sarcoma, which metastasized to regional and bone sites 23. The patient had previously undergone surgery, radiotherapy and chemotherapy before undergoing [^68^Ga]Ga-FAPI-46 PET/CT imaging followed by [^177^Lu]Lu-FAPI-46 therapy. The treatment consisted of four cycles, totaling a cumulative dose of 8.5 GBq. Posttherapy imaging, evaluated according to the RECIST 1.1 criteria, revealed PD. The patient experienced toxicity, including Grade 1 thrombocytopenia and leukopenia, as well as Grade 3 anemia. We highlight that this is the first reported use of [^177^Lu]Lu-FAPI-46 in a pediatric patient 23.

Banihashemian *et al.* presented a 67-year-old male with leiomyosarcoma that had metastasized to the lymph nodes and the chest wall 30. The patient received [^177^Lu]Lu-FAPI-2286 therapy following the failure of previously received chemotherapy and radiotherapy. The treatment involved 4 cycles with a cumulative dose of 23 GBq. Post-therapeutic scans were conducted at 1 hour, 24, 48, and 72 hours, as well as 8 days postinjection, which was in accordance with the [^68^Ga]Ga-FAPI findings. The imaging response revealed PD, and the patient experienced no adverse events, with no significant changes in serial hematological tests, including blood cell, renal, and liver function tests 30.

Banihashemian *et al.* subsequently investigated the effects of [^177^Lu]Lu-FAPI-2286 in five patients (3 males and 2 females) with various types of sarcoma back in 2024 29. The sarcoma types included mediastinal leiomyosarcoma, thyroid sarcoma, alveolar soft sarcoma, pleomorphic cell sarcoma, and neurofibrosarcoma. The patients, aged between 28 and 67 years, had previously undergone various treatments, including surgery, chemotherapy, and radiotherapy. Each patient was evaluated via [^68^Ga]Ga-FAPI-2286 PET/CT before receiving [^177^Lu]Lu-FAPI-2286 therapy. All five patients received four cycles of treatment, with cumulative doses ranging from approximately 26.4 to 29.6. GBq (ranging from 6.6 to 7.4 GBq per cycle). The study revealed significant clinical improvements, particularly in patients' physical capacity, with a marked reduction in pain for some individuals. According to the RECIST 1.1 criteria, post-therapy imaging assessments revealed a PR in four patients, whereas one patient with pleomorphic cell sarcoma had a PD. Importantly, no grade 3 or 4 toxicities or clinically significant adverse effects were reported 29.

**Figure 4:**
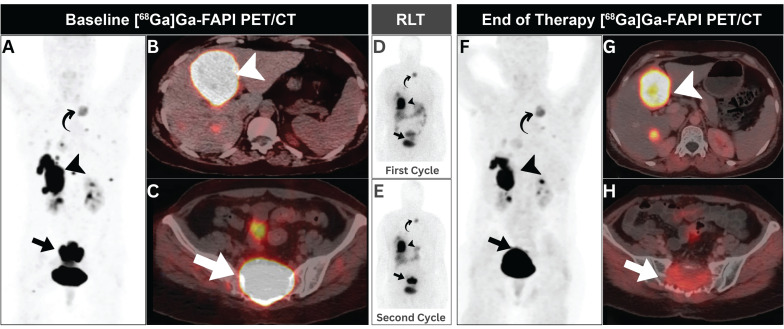
A 49-year-old woman with metastatic solitary fibrous tumor. (A-C) Initial [^68^Ga]Ga-FAPI PET/CT revealed intense uptake in hepatic (arrowheads), and sacral (arrows) metastatic lesions with minimal expression in other metastatic lesions elsewhere (curved arrow). (D, E) Post-treatment scintigraphy demonstrated satisfactory [^177^Lu]Lu-FAPI localization without side effects. (F-H) Follow-up [^68^Ga]Ga-FAPI PET/CT showed regression of sacral and hepatic lesions (arrows), with otherwise stable previous small metastatic lesions elsewhere (curved arrow). Adapted with permission from reference 38, copyright: © 2023 Indian Journal of Nuclear Medicine. Published under creative commons (License: CC BY-NC-SA 4.0).

Luthra *et al.* presented a 49-year-old female with a metastatic solitary fibrous tumor 38. The patient was treated with two cycles of [^177^Lu]Lu-FAPI (a specific formulation not mentioned), totaling 14.8 GBq. At 6-9 weeks after therapy, there was a decrease in tumor size and FAPI expression, indicating that the patient experienced partial pain relief after the first cycle and significant pain reduction after the second cycle. No significant hematological toxicity was observed between cycles (Figure 4) 38.

Yang *et al.* 2024 described the treatment of a 57-year-old female with a solitary fibrous tumor that had spread to bones, kidneys, lungs, stomach, and pancreas 42. The patient underwent transurethral bladder resection, followed by chemotherapy and immunotherapy as part of their treatment regimen for bladder cancer. Moreover, he received one cycle of [^177^Lu]Lu-FAPI-2286 at a dose of 7.4 GBq. The imaging response at 6-9 weeks posttherapy was a PR with a significant reduction in tumor size and FAPI expression, and the treatment was well tolerated with no adverse effects 42.

### Gynecological malignancies

Two studies investigated the use of [^177^Lu]Lu-FAPI in four female patients with gynecological cancers. Assadi investigated the use of [^177^Lu]Lu-FAPI-46 in three women with advanced cancers: one with cervical cancer and two with ovarian cancer 23. These patients, aged 44 to 47 years, had cancer spread to various organs. Before treatment, [^68^Ga]Ga-FAPI-46 PET/CT scans were performed. The cervical cancer patient received two cycles of [^177^Lu]Lu-FAPI-46 therapy with a total dose of 6.66 GBq. Follow-up imaging revealed PD. The two ovarian cancer patients received [^177^Lu]Lu-FAPI-46 at different dosages: one received 3.7 GBq over one cycle, and the other received 10 GBq over three cycles. Follow-up imaging revealed SD results in both patients. None of the three patients experienced any significant side effects from [^177^Lu]Lu-FAPI-46 treatment 23.

Baum *et al.* reported the use of [^177^Lu]Lu-FAP-2286 in a 50-year-old woman with ovarian cancer 32. The cancer had spread to other areas, underwent surgery and received chemotherapy and immunotherapy. The patient received two cycles of [^177^Lu]Lu-FAP-2286 therapy with a total dose of approximately 5.8 GBq. Follow-up imaging revealed PD, but the patient did not experience any significant side effects 32.

### Other malignancies

Assadi explored the utility of [^177^Lu]Lu-FAPI-46 was assessed in a 28-year-old male with liver, peritoneal, lymph node, and bone metastases from a round-cell tumor 23. This patient received two cycles of [^177^Lu]Lu-FAPI-46 totaling 6.66 GBq. The imaging response indicated SD, and no toxicity was reported 23.

Barashki *et al.* reported a male patient with multiple endocrine neoplasia type 2A (MEN2A) syndrome-associated pheochromocytoma/paraganglioma who had metastases in the liver, lymph nodes, bones, and lungs 31. After surgery, radiotherapy and chemotherapy, the patient was imaged via [^68^Ga]Ga-FAPI-46 PET/CT before receiving one cycle of [^177^Lu]Lu-FAPI-46 at a dose of 7.4 GBq. The patient experienced resolution of abdominal pain shortly after treatment, with no adverse events reported. However, posttherapy sequential imaging performed daily for 3 days suggested relatively rapid clearance of the radiolabeled FAPI from metastatic lesions. Unfortunately, imaging response by PET/CT, CT or MRI was not performed or reported in the study 31.

## [^90^Y]Y-FAPI RLT: systematic review

The remaining four studies utilized a total of 103 [^90^Y]Y-FAPI RLT cycles offered at a median active dose of 6.7 GBq/cycle 46-49. These studies explored [^90^Y]Y-FAPI RLT utility in various cancer types encompassing a total of 42 patients (15 males and 27 females). Table 3 outlines etiology-based analysis of studies utilizing [^177^Lu]Lu-FAPI RLT.

### Gastroenteropancreatic malignancies

Two studies evaluated the use of [^90^Y]Y-FAPI-46 in patients with Gastroenteropancreatic malignancies, encompassing six cases of pancreatic ductal adenocarcinoma (PDAC) and one case of gastric cancer. Ferdinandus *et al.* conducted a retrospective case series, including nine patients, of whom three had PDAC and six had metastatic sarcomas 47. The PDAC subgroup comprised exclusively female patients with a median age of 58 years, exhibiting extensive metastatic disease. Each patient received a single cycle of [^90^Y]Y-FAPI-46 following multiple lines of prior systemic therapy. Pre-treatment imaging with [^68^Ga]Ga-FAPI PET/CT was performed to determine [^90^Y]Y-FAPI-46 eligibility. Administered [^90^Y]Y-FAPI-46 doses ranging from 3 to 3.8 GBq were employed. Treatment response, evaluated using RECIST 1.1 and PERCIST criteria, demonstrated PD in two patients. The third patient experienced clinical and biochemical progression but did not undergo molecular imaging evaluation. All three patients experienced grade 3/4 toxicities, including thrombocytopenia and hepatotoxicity 47.

Concurrently, Fendler *et al.* reported on 21 patients treated with [^90^Y]Y-FAPI-46, including three female PDAC patients with a median age of 59 years 46. All patients had advanced metastatic disease affecting multiple organs and had undergone a median of three local therapies and four systemic treatment lines prior to FAPI RLT. Pre-therapeutic imaging was performed using [^68^Ga]Ga-FAPI-46 PET/CT. Within the PDAC subgroup, extensive neoplastic disease was evident on molecular imaging, involving the pancreas, liver, lungs, lymph nodes, and bones. All patients had previously received multiple systemic therapies, including various chemotherapy regimens and targeted agents such as Olaparib and Afatinib. PDAC patients received a total of 7 [^90^Y]Y-FAPI-46 with a median active dose of 7.3 GBq. Treatment response assessment indicated SD in one patient, while the other two demonstrated PD. No RLT-related adverse effects were noted 46. Additionally, Fendler *et al.* described a 38-year-old female with metastatic gastric cancer involving the stomach, lymph nodes, liver, peritoneum, and spleen who underwent a single cycle of [^90^Y]Y-FAPI-46 dosed at 7.4 GBq after prior multiple systemic chemo- and immunotherapy regimens 46. Despite receiving RLT guided by positive [^68^Ga]Ga-FAPI-46 PET/CT imaging, follow-up assessments revealed PD. No RLT-related adverse effects were observed 46.

### Genitourinary malignancies

Fendler *et al.* also reported on an 81-year-old male patient with advanced prostate cancer involving both the prostate gland and extensive skeletal metastases 46. The patient had undergone multiple prior therapies, including androgen deprivation therapy, docetaxel chemotherapy, enzalutamide, and [^223^Ra]RaCl2. Pre-treatment [^68^Ga]Ga-FAPI-46 PET/CT imaging confirmed sufficient radiotracer uptake, prompting the administration of a single 7.4 GBq [^90^Y]Y-FAPI-46 therapy cycle. Subsequent [^68^Ga]Ga-FAPI-46 PET/CT imaging demonstrated PD, suggesting limited therapeutic efficacy in this case. No RLT-related adverse effects were observed 46.

### Mesenchymal malignancies

Two studies investigated the application of [^90^Y]Y-FAPI-46 in patients with sarcomas, collectively including 33 patients across various subtypes. Ferdinandus *et al.* evaluated [^90^Y]Y-FAPI-46 in six patients with metastatic sarcomas, each representing a different histological subtype: osteosarcoma, chordoma, fibrosarcoma, gastrointestinal neuroectodermal sarcoma, conventional chondrosarcoma, and spindle cell sarcoma 47. All patients presented with extensive metastatic disease affecting multiple organs. Pre-treatment [^68^Ga]Ga-FAPI PET/CT imaging was performed, and treatment response was assessed using bremsstrahlung scintigraphy and post-therapy [^68^Ga]Ga-FAPI PET/CT imaging. Administered doses ranged from 3.5 GBq to 18.3 GBq over one to three treatment cycles. Response assessment using RECIST 1.1 and PERCIST criteria revealed diverse outcomes: PD in three patients, SD in one patient, and PR in one patient. Grade 3/4 thrombocytopenia occurred in one patient with osteosarcoma 47.

Fendler *et al.* examined seven patients with metastatic sarcomas treated with [^90^Y]Y-FAPI-46 46. The cohort included patients diagnosed with fibrosarcoma, gastrointestinal neuroectodermal sarcoma, chondrosarcoma, spindle cell sarcoma, leiomyosarcoma, chondroma, and osteosarcoma, all presenting with extensive metastatic disease. Pre-therapeutic [^68^Ga]Ga-FAPI-46 PET/CT imaging was performed. The patients received a total of 18 cycles of [^90^Y]Y-FAPI-46, with a median dose of 7.4 GBq. Treatment responses varied, with SD observed in three patients and PD documented in three cases. Four events of high-grade thrombocytopenia were recorded according to CTCAE version 5.0 46.

Fendler *et al.* also examined a subgroup of nine patients with metastatic solitary fibrous tumors, evaluating the therapeutic application of [^90^Y]Y-FAPI-46 in patients presenting with extensive metastatic disease involving multiple organ systems 46. All patients had undergone multiple lines of systemic therapies. The patients received a total of 20 cycles of [^90^Y]Y-FAPI-46, with a median dose of 7.4 GBq. Treatment response assessment using RECIST 1.1 and PERCIST criteria revealed SD in five patients, PD in two, and PR in one patient, with no data available for two patients. No RLT-induced toxicities were reported 46.

Hamacher *et al.* conducted a prospective observational study, evaluating the therapeutic efficacy of [^90^Y]Y-FAPI-46 in patients with solitary fibrous tumors 48. Of 19 enrolled patients, 11 received therapy (median age: 61 years, range: 36-85; 8 females, 3 males). Pre-treatment [^68^Ga]Ga-FAPI-46 PET/CT imaging confirmed eligibility. A total of 34 therapy cycles were administered (median: 3 cycles, interquartile range: 2 cycles). Treatment response assessment indicated PR in three patients (27%) and SD in six patients (55%), resulting in a disease control rate of 82%. No adverse events or toxicities were reported 48.

### Multiple primary neoplasia

Rathke *et al.* presented an intriguing case involving a female patient with multiple primary neoplasms, initially diagnosed with breast malignancy followed by metachronous colorectal cancer 49. The patient presented with extensive metastatic disease, including peritoneal metastases from colorectal cancer and nodal, osseous, and hepatic metastases from breast cancer. Following prior chemotherapy, the patient underwent FAPI RLT. Four cycles of [^90^Y]Y-FAPI-46 RLT were administered (cumulative dose: 35.5 GBq). Post-treatment bremsstrahlung imaging using a gamma camera confirmed radiotracer distribution. Subsequent evaluation revealed a heterogeneous response: PD disease was observed in breast cancer-related lesions, while colorectal metastases demonstrated complete remission. The authors did not report on toxicity or adverse events in this case 49.

## Innovative therapy approaches

**Figure 5:**
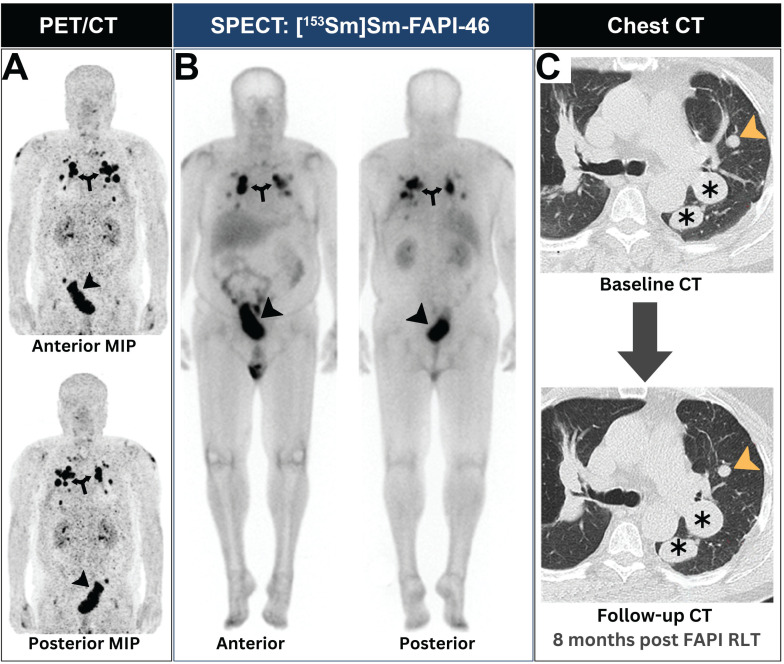
A patient with lung sarcoma achieved successful neoplastic disease control after receiving FAPI RLT. (A) Baseline PET revealed intense [^68^Ga]Ga-FAPI expression at both pulmonary (arrows) and pelvic lesions (arrowheads). (B) SPECT imaging showed adequate scintigraphic localization of [^153^Sm]Sm-FAPI-46 within all neoplastic lesions (annotations). (C) After two [^153^Sm]Sm-FAPI-46 cycles and a single [^90^Y]Y-FAPI-46 cycle, the patient achieved stable disease control in all lesions as evident by chest CTs (annotations). Images were adapted with permission from reference 45, copyright: © 2021 Springer Nature. Published under creative commons (License: CC BY 4.0).

Kratochwil *et al.* employed a dual radionuclide FAPI radioligand therapy (RLT) approach in a patient with metastatic lung sarcoma 45. The treatment regimen consisted of three [^153^Sm]Sm-FAPI-46 cycles followed by a single [^90^Y]Y-FAPI-46 cycle. After exhausting multiple lines of systemic therapy, the patient underwent [^68^Ga]Ga-FAPI-46 PET/CT imaging to determine eligibility for FAPI RLT. Upon confirmation of eligibility, the patient received two [^153^Sm]Sm-FAPI-46 cycles with a cumulative activity of 20 GBq, followed by a single 8 GBq [^90^Y]Y-FAPI-46 cycle. Posttreatment SPECT examination revealed adequate scintigraphic localization within the tumor of interest. The treatment was well-tolerated, with no observed toxicities 45. Notably, this therapeutic approach resulted in stable disease for a total of 8 months, as evidenced by follow-up CT imaging (Figure 5).

Helisch *et al.* recently investigated a fractionated dose regimen utilizing [^213^Bi]Bi-FAPI-46 RLT in a cohort of six cancer patients 44. The study enrolled six patients (four women and two men) with progressive [^68^Ga]Ga-FAPI-avid metastatic solid tumors, including three colon cancer, one anal cancer, one breast cancer, and one prostate cancer. Age of included patients ranged from 16 years to 77 years. All patients had exhausted conventional therapies (surgery, chemotherapy, immunotherapy, and radiotherapy) and received a mean administered activity of 1.6 MBq of [^213^Bi]Bi-FAPI-46, fractionated into 5 to 12 intravenous applications over a period of up to 107 hours. Four of the patients also received concurrent pembrolizumab immunotherapy. This includes two patients diagnosed with colonic cancer, one patient with anal cancer, and one patient with breast cancer. Response assessments were conducted using post-treatment [^18^F]FDG and [^68^Ga]Ga-FAPI-46 PET/CT scans for four and five patients, respectively. [^213^Bi]Bi-FAPI-46 was well-tolerated in all patients without adverse side effects. Post-treatment imaging evaluation revealed that four patients (three colon cancer, one breast cancer) failed to achieve disease control and experienced PD. Disease control was achieved in the form of PR in a patient with prostate cancer and SD in a patient with anal squamous cell carcinoma 44.

## Discussion

To the best of our knowledge, this systematic review is the first to comprehensively highlight the use of FAPI RLT as a novel and promising therapeutic approach in the field of oncology. Our analysis covers 27 studies, which together include a cohort of 144 patients diagnosed with a range of malignancies. With regards to clinical efficacy, disease control was evident in all clinical investigations ranging from 18.2%-83.3% 23, 25, 29, 32, 34, 41, 44, 46-48. Post FAPI RLT, nine out of 17 reported cases involving sarcoma, lung adenocarcinoma, lung squamous carcinoma, invasive breast carcinoma, refractory differentiated thyroid cancer, medullary thyroid cancer, glioblastoma multiforme, and rhabdoid meningioma achieved disease control.

Importantly, the safety profile of FAPI RLT also appears favorable. Among the 144 patients treated, only 14 patients experienced grade 3 or 4 toxicities, suggesting that the therapy is generally well tolerated and has a relatively low incidence of severe adverse effects. This finding further underscores the potential of FAPI RLT not only as an efficacious treatment but also as a safe option for patients with advanced cancers.

None of the included studies directly compared FAPI RLT therapy with standard treatments, underscoring a significant gap in the current research. Moreover, most patients in these studies had undergone extensive pretreatment with surgery, chemotherapy, immunotherapy, and/or radiotherapy prior to receiving FAPI RLT, often under compassionate use circumstances. Therefore, investigating the efficacy of FAPI RLT in treatment-naïve patients could offer valuable insights into its potential effectiveness at earlier stages of cancer treatment.

The predictive capability of value of baseline [^68^Ga]Ga-labeled FAPI PET for [^177^Lu]Lu-FAPI RLT efficacy remains an active area of research. Although [^68^Ga]Ga-FAPI PET exhibits high sensitivity and specificity in detecting FAP-expressing tumors 14, its capacity to forecast therapeutic outcomes with corresponding [^177^Lu]Lu-FAPI agents is multifaceted and not yet fully elucidated. FAPI-targeted therapeutic agents face significant challenges that limit their treatment efficacy. The primary drawback is the rapid washout of these compounds from tumor tissues, diminishing their therapeutic potential 50. This rapid clearance is particularly problematic for radionuclides with longer half-lives, as the physical decay does not align with the biological half-life of the radiopharmaceutical in the tumor. The mismatch between tumor retention time and radionuclide half-life results in suboptimal radiation dose delivery to the target tissue. For instance, [^177^Lu]Lu-FAPI-46 and [^225^Ac]Ac-FAPI-46 demonstrated low tumor uptake at 3- and 24-hour post-injection, limiting their therapeutic efficacy 51. This rapid clearance not only reduces the anti-tumor effect but also increases radiation exposure to non-target organs, particularly the kidneys.

To address these issues, researchers have explored various strategies, including the development of multimeric FAPI molecules and the incorporation of albumin binders 26, 27, 34. The prolongation of FAP binding molecule circulating half-life with an albumin binder may improve cumulative uptake in tumor as was previously seen with a recombinant anti-FAP antibody, and which may enhance delivered dose and potential therapeutic effect (5). Similarly, improving affinity for FAP and uptake in tumor may also result in higher dosimetry in tumor. While these approaches can enhance tumor uptake and retention, they also present a double-edged sword. Prolonged circulation time may increase tumor accumulation but simultaneously elevate non-specific uptake in normal tissues, potentially leading to increased toxicity 52. Balancing treatment efficacy and side effects requires careful optimization of pharmacokinetics and pharmacodynamics. Personalized dosimetry, fractionated dosing regimens, and the exploration of radionuclides with shorter half-lives may help achieve this balance 53. Additionally, combining FAP-targeted therapies with other treatment modalities targeting tumor cells directly could potentially improve overall therapeutic outcomes 53.

A significant observation from this review is presented in two studies 28, 42, which investigated the potential of combining [^177^Lu]Lu-FAPI therapy with chemotherapy and/or targeted immunotherapy. Notably, both patients in these studies exhibited positive clinical outcomes, with one achieving a complete response and the other a partial response to the combined treatment approach. This promising outcome highlights an important direction for future research, emphasizing the necessity for further investigation into the synergistic effects of FAPI therapy when combined with chemotherapy and immunotherapy to optimize patient outcomes.

Moreover, FAP-targeted radiopharmaceuticals incorporating shorter half-life radionuclides such as [^67^Cu]Cu, [^211^At]At, [^188^Re]Re, and [^153^Sm] might offer several potential advantages for FAP-targeted RLT 54. They may provide the required balance between physical decay and biological clearance, potentially improving the therapeutic index. Additionally, they offer the ability to deliver higher initial radioactivity to the tumor, which may enhance treatment efficacy. Further benefits include reduced radiation exposure to non-target tissues, potentially lower toxicity, and shorter patient isolation periods 54.

Regarding the selection of alpha or beta emitters for FAP-targeted RLT, most studies have utilized beta emitters, such as ^177^Lu or ^90^Y. Beta emitters are generally considered more effective than alpha emitters due to their cross-fire effect, which allows radiation from cancer-associated fibroblasts (CAFs) to impact surrounding cancer tissues. However, alpha emitters, with their high linear energy transfer (LET), induce efficient DNA double-strand breaks and can ablate cancer cells more effectively than beta emitters. In a preclinical study comparing [^177^Lu]Lu-FAPI-46 and [^225^Ac]Ac-FAPI-46, [^225^Ac]Ac-FAPI-46 demonstrated a better early therapeutic response compared to [^177^Lu]Lu-FAPI-46 51. However, tumor regrowth occurred more quickly with [^225^Ac]Ac-FAPI-46, potentially due to the limited range of alpha particles, which may leave regions of the tumor insufficiently irradiated. Tandem therapy combining alpha and beta emitters could be a promising approach and warrants evaluation in prospective clinical trials.

Regarding posttreatment evaluation, [^68^Ga]Ga-FAPI PET alone is insufficient due to the dynamic nature of FAPI expression following treatment, which may not accurately reflect tumor volumes 29. Therefore, a comprehensive rather than selective assessment approach is necessary. This includes a multifaceted evaluation of clinical, biochemical, and imaging metrics 55. The evaluation should focus on symptom progression, patient-specific functional status, and patient-reported outcomes. Tumor-specific biomarkers can provide quantitative insights into disease burden. Complementary imaging techniques like CT or MRI are essential for assessing anatomical alterations. Moreover, dosimetry studies offer valuable data on tumor and organ exposure 55. Furthermore, rigorous monitoring of hematological, renal, and hepatic parameters is crucial for safety profiling. Extended follow-up periods are necessary to determine overall and progression-free survival rates 17. While this systematic review presents post-treatment imaging response profiles from the included studies, it is constrained by the limited scope of post-treatment evaluation methodologies employed in the original research 23, 25, 29, 32, 34, 41, 44, 46-48. The existing literature provides only a short-term snapshot of observations following FAPI RLT. Consequently, forthcoming studies should prioritize the implementation of more comprehensive evaluation approaches to elucidate a more thorough understanding of treatment outcomes.

Several ongoing clinical trials are exploring the potential of FAPI RLT across various cancer types. These trials aim to evaluate the safety, efficacy and dosimetry of different FAPI variants, including [^177^Lu]Lu-DOTA-FAPI, [^177^Lu]Lu-EB-FAPI and [^177^Lu]Lu-PNT6555 56. There is also similar emphasis on recruiting clinical trials to investigate and evaluate alternative forms of [^177^Lu]Lu-FAP-based targeting therapies, rather than employing FAPI (Table 4).

It's important to indicate that this review has several limitations. First, the review lacks data on other certain cancer subtypes known to exhibit high target-to-background ratios (TBRs) in [^68^Ga]Ga-FAPI PET/CT imaging, such as hepatobiliary tumors, gastric cancer and mesothelioma. Furthermore, significant variability exists across the included studies, with marked differences in patient populations, cancer types, and study methodologies, which complicates the ability to conduct a unified analysis. Additionally, many of the studies are limited to case reports or interesting imaging cases, while the clinical trials included lack both randomization and control groups. The short follow-up periods and small sample sizes in these studies further restrict the ability to evaluate long-term efficacy and validation comprehensively. Consequently, it is imperative to conduct additional studies involving larger sample sizes and extended follow-up durations to enhance our understanding of the intervention's efficacy and long-term safety across various types of cancer.

## Conclusion

This systematic review presents a comprehensive analysis of the use of FAPI RLT across various cancer types. The findings revealed that FAPI RLT demonstrates potential therapeutic efficacy in a wide range of malignancies. Imaging responses vary, with partial response and stable disease being common outcomes. The safety profile of FAPI RLT is promising, with minimal reported grade 3-4 toxicities and no life-threatening adverse events. However, further research with larger cohorts and longer follow-up periods is needed to better understand its efficacy and long-term safety across different cancer types.

## Supplementary Material

Supplementary table.

## Figures and Tables

**Table 1:** General characteristics of the included studies

Article	Country	Study type	Cancer Type	Patients (Gender)	Age	FAPI RLT Agent
Ballal *et al.*, 2021 24	IN	CR	BC	1 F	31	[^177^Lu]Lu-DOTA-SA-FAPI
Assadi *et al.*, 2021 23	IR	OR	Various	18 (10 F, 8 M)	50.5*	[^177^Lu]Lu-FAPI-46
Rathke *et al.*, 2021 49	US	CR	SN (CRC+ BC)	1 (1 F)	-	[^90^Y]Y-FAPI-46
Kratochwil *et al.*, 2021 45	DE	CR	LC	1 (1 M)	-	[^153^Sm]Sm-FAPI-46 + [^90^Y]Y-FAPI-46
Ferdinandus *et al.*, 2022 47	DE	OR	Various	9 (5 F, 3 M)	57*	[^90^Y]Y-FAPI-46
Fendler *et al.*, 2022 46	US	OR	Various	21 (13 F, 8 M)	61*	[^90^Y]Y-FAPI-46
Ballal *et al.*, 2022 25	IN	OP	RRDTC	15 (11 F, 4 M)	55	[^177^Lu]Lu-DOTAGA-(SA-FAPi)_2_
Kaghazchi *et al.*, 2022 36	IR	CR	PDAC	1 F	52	[^177^Lu]Lu-FAPI-46
Baum *et al.*, 2022 32	DE	OR	Various	11 (6 F, 5 M)	61*	[^177^Lu]Lu-FAP-2286
Barashki *et al.*, 2022 31	IR	CR	MEN2A	1 M	-	[^177^Lu]Lu-FAPI-46
Fu *et al.*, 2022 35	CN	CR	NPC	1 M	25	[^177^Lu]Lu-FAPI-46
Ballal *et al.*, 2022 26	IN	CR	MTC	1 M	56	[^177^Lu]Lu-DOTAGA-(SA-FAPi)_2_
Fu *et al.*, 2022 33	CN	CR	RRDTC	1 M	34	[^177^Lu]Lu-FAPI-46
Fu *et al.*, 2023 34	IN	OP	RRDTC	12 (8 M, 4 F)	52*	[^177^Lu]Lu-EB-FAPI ([^177^Lu]Lu-LNC1004)
Luthra *et al.*, 2023 38	IN	CR	SFT	1 F	49	[^177^Lu]Lu-FAPI (not specified)
Li *et al.*, 2023 37	CN	CR	UBC	1 M	73	[^177^Lu]Lu-FAP-2286
Rao *et al.*, 2023 39	CN	CR	SLC	1 M	49	[^177^Lu]Lu-FAP-2286
Yadav *et al.*, 2024 41	IN	OR	BC	19 (18 F, 1 M)	45.5*	[^177^Lu]Lu-DOTAGA-(SA-FAPi)_2_
Hamacher *et al.*, 2024 48	DE	OP	SFT	11 (8 F, 3 M)	61	[^90^Y]Y-FAPI-46
Banihashemian *et al.*, 2024 30	IR	CR	LMS	1 M	67	[^177^Lu]Lu-FAPI-2286
Banihashemian *et al.*, 2024 28	IR	CR	BC	1 F	42	[^177^Lu]Lu-FAPI-2286
Yang *et al.*, 2024 43	CN	CR	LAC	1 M	56	[^177^Lu]Lu-FAP-2286
Yang *et al.*, 2024 42	CN	CR	SFT (Bone)	1 F	57	[^177^Lu]Lu-FAPI-2286
Ballal *et al.*, 2024 27	IN	CR	GBM	1 F	52	[^177^Lu]Lu-DOTAGA-Glu-(FAPI)_2_
Wan *et al.*, 2024 40	CN	CR	RM	1 M	43	[^177^Lu]Lu-FAPI-2286
Banihashemian *et al.*, 2024 29	IR	OP	Sarcoma	5 (3 M, 2 F)	57*	[^177^Lu]Lu-DOTAGA-Glu-(FAPI)_2_
Helisch *et al.*, 2024 44	DE	OR	Various	6 (4 F, 2 M)		[^213^Bi]Bi-FAPI-46

*, Median Values; BC, Breast Cancer; CN, China; CR, Case Report; CRC, Colorectal cancer; DE, Germany; F, Female; G1, Grade 1; G2, Grade 2; G3, Grade 3; G4, Grade 4; GBM, Glioblastoma Multiforme; IN, India; IR, Iran; LAC, Lung Adenocarcinoma; LC, Lung Cancer; LMS, Leiomyosarcoma; M, Male; MEN2A, Multiple Neuroendocrine Neoplasia 2A; MTC, Medullary Thyroid Cancer; n, Number; NPC, Nasopharyngeal Carcinoma; NR, Not reported; OP, Original Prospective Study; OR, Original Retrospective Study; PDAC, Pancreatic ductal adenocarcinoma; RM, Rhabdoid Meningioma; RRDTC, Radioiodine-refractory Differentiated Thyroid Cancer; SFT, Solitary Fibrous Tumor; SLC, Squamous lung Cancer; SN, Synchronous neoplasms; UBC, Urinary Bladder Cancer

**Table 2:** Etiology-based Analysis of studies exploring [^177^Lu]Lu-FAPI

Cancer type		Number of patients
Head and neck malignancies	CNS	2
	Nasopharyngeal	1
	Thyroid	30
Thoracic malignancies	Breast	30
	Lung	3
Gastroenteropancreatic malignancies	Pancreatic	8
	Colorectal	4
Genitourinary malignancies	Urinary bladder	1
	Prostate	1
Gynecological malignancies	Ovarian	3
	Cervical	1
Mesenchymal malignancies	Sarcoma	7
	SFT	2
Rare tumors	Round cell tumor	1
	MEN2A	1
Total		80

CNS, Central Nervous System; MEN2A, Multiple Endocrine Neoplasia Type 2A; SFT, Solitary Fibrous Tumor.

**Table 3:** Etiology-based Analysis of studies exploring [^90^Y]Y-FAPI. BC, Breast cancer; CRC, Colorectal cancer; SFT, Solitary fibrous tumor.

Cancer type		Number of patients
Gastroenteropancreatic	Pancreatic	6
	Gastric	1
Genitourinary malignancies	Prostate	1
Mesenchymal malignancies	Sarcoma	33
Multiple primary neoplasia	BC + CRC	1

**Table 4:** Selection of pending clinical trials exploring therapeutic utilities of [^177^Lu]Lu-FAPI in various cancers

Trial Number	Phase	Neoplasms	Agent	Country
NCT04849247	I	Various	[^177^Lu]Lu-DOTA-FAPI	China
NCT05410821	I	Metastatic RRDTC	[^177^Lu]Lu-DOTA-EB-FAPI	China
NCT05400967	I	Metastatic Solid Tumors	[^177^Lu]Lu- EB-FAPI	China
NCT05963386	I	Refractory Solid Tumors	[^177^Lu]Lu-DOTA-EB-FAPI	China
NCT06081322	I	Advanced Pancreaticobiliary	[^177^Lu]Lu- EB-FAPI	China
NCT05432193	I	Various	[^177^Lu]Lu-PNT6555	USA and Canada
NCT06636617	I	Various	[^177^Lu]Lu-JH04	China
NCT04939610	I/II	Various	[^177^Lu]Lu-FAP-2286	USA
NCT06640413	I	Various	[^177^Lu]Lu-OncoFAP-23	Italy
NCT06197139	I	Various	[^177^Lu]Lu-XT117	China
NCT06638034	I	Various	[^177^Lu]Lu-FAPI-RGD	China

RRDTC, Radioiodine-refractory Differentiated Thyroid Cancer; USA, United States of America.

## Abbreviations

AEs: Adverse Events
CAF: Cancer Associated Fibroblast
CgA: Chromogranin A
CR: Complete Response
CT: Computed Tomography
CTCAE: Common Terminology Criteria for Adverse Events
DLT: Dose-Limiting Toxicity
ECOG: Eastern Cooperative Oncology Group
FAP: Fibroblast Activation Protein
FAPI: Fibroblast Activation Protein Inhibitor
MEN2A: Multiple Endocrine Neoplasia Type 2A
MRI: Magnetic Resonance Imaging
NCI: National Cancer Institute
PD: Progressive Disease
PERCIST: PET Response Criteria in Solid Tumors
PET: Positron Emission Tomography
PR: Partial Response
PRISMA: Preferred Reporting Items for Systematic Reviews and Meta-Analyses
RAI: Radioactive Iodine
RECIST 1.1: Response Evaluation Criteria in Solid Tumors version 1.1
RLT: Radioligand Therapy
ROBINS-I: Risk of Bias in Non-randomized Studies - of Interventions
SD: Stable Disease
TME: Tumor Microenvironment
